# Persistent Spillback of Bovine Tuberculosis From White-Tailed Deer to Cattle in Michigan, USA: Status, Strategies, and Needs

**DOI:** 10.3389/fvets.2018.00301

**Published:** 2018-11-29

**Authors:** Kurt C. VerCauteren, Michael J. Lavelle, Henry Campa

**Affiliations:** ^1^National Wildlife Research Center, USDA/APHIS/Wildlife Services. Fort Collins, CO, United States; ^2^Department of Fisheries and Wildlife, Michigan State University, East Lansing, MI, United States

**Keywords:** bovine tuberculosis, cattle, disease, *Odocoileus virginianus*, transmission, spillback, spillover, white-tailed deer

## Abstract

Free-ranging white-tailed deer (*Odocoileus virginianus*) are believed to be a self-sustaining reservoir for bovine tuberculosis (bTB) in northeastern Lower Michigan, USA. Although a comprehensive control program is in place and on-farm mitigation strategies to curtail bTB transmission between cattle and deer have been implemented for over a decade, cattle and deer continue to become infected with the disease. Thus, renewed motivation to eradicate bTB is needed if that is truly the goal. Recurrent detection of bTB in cattle in the region is of mounting concern for state and federal agricultural agencies, producers, and wildlife managers. Current on-farm mitigation efforts include fencing and refined cattle feeding and watering practices. Liberal removal of antlerless deer through hunter harvest and disease control permits (DCPs) issued to cattle producers and agency sharp shooters have also been ongoing. Although these strategies have merit and efforts to reduce prevalence in deer and occurrence of positive farms are elevated, additional actions are needed. Heightened management actions to combat bTB in deer could include deer vaccination programs, strategic habitat manipulations to redistribute deer from farms, and precision removal of deer in proximity to high-risk farms. Foundational research to address development and delivery of vaccine to free-ranging deer is complete. Strategic management and habitat manipulation could reduce and disperse local concentrations of deer while better meeting wildlife, forestry, and agricultural goals. The responses of local deer populations to targeted removal of individuals are generally understood and there is potential to reduce deer activity around agricultural operations while allowing them to persist nearby on natural foods. We summarize the history and progress to date, discuss the realized merit of novel management strategies, and suggest options to rid deer and cattle in Michigan of bTB.

## Key concepts

**Integrated disease management:** employing a variety of proven strategies simultaneously to most efficiently achieve management objectives.

**Mitigation measures to protect cattle:** specific actions taken to reduce potential for direct and indirect transmission of *M. bovis* from wildlife to cattle.

**Management strategies for deer:** specific actions designed to reduce potential for maintaining disease within free-ranging deer such as using hunters or professional sharpshooters to reduce deer numbers and eliminating the provisioning of anthropogenic food sources with the intent of attracting and maintaining deer concentrations.

**Negative impacts of supplemental feeding and baiting:** anthropogenic feeding leads to artificially high and concentrated populations of wildlife which in turn increases disease transmission risk and prevalence.

**Setting realistic goals:** developing a documented and well-informed formal strategy designed to reach a common and achievable goal.

**Public support, political will:** varying stakeholder motivations must be considered, reconciled and presented to decision makers so they can empower the pursuit of common goals.

## Introduction

### History of bovine tuberculosis in michigan, USA

Bovine tuberculosis (bTB), caused by the *Mycobacterium bovis* (*M. bovis*) bacterium was historically a disease among cattle that spilled over into free-ranging wildlife where it persists (1–3). Bovine tuberculosis is a threat to national and international beef and dairy markets. There are currently more than 13,000 cattle producers maintaining >1.1 million cattle in Michigan. The United States Department of Agriculture (USDA) has 5 levels of zoning regarding bTB status that states, or zones within states, fall into regarding presence of bTB infection in cattle ranging from 1 with no apparent prevalence in cattle and bison (*Bison bison*) to 5 with an unknown or ≥ 0.5% herd prevalence. The 5 levels include: (1) Accredited-free zone (“TB free”), (2) Modified accredited advanced zone (MAAZ), (3) Modified accredited zone (MAZ), (4) Accredited preparatory zone, and (5) Non-accredited zone. Zoning enables agencies to tailor surveillance and management strategies relative to regional disease prevalence and potential risk of spread (4). The continual appearance of bTB in livestock facilities in Michigan annually keeps the zoning status of the state at risk while maintaining producer's ability to engage in national and international markets (5).

**Figure 1:**
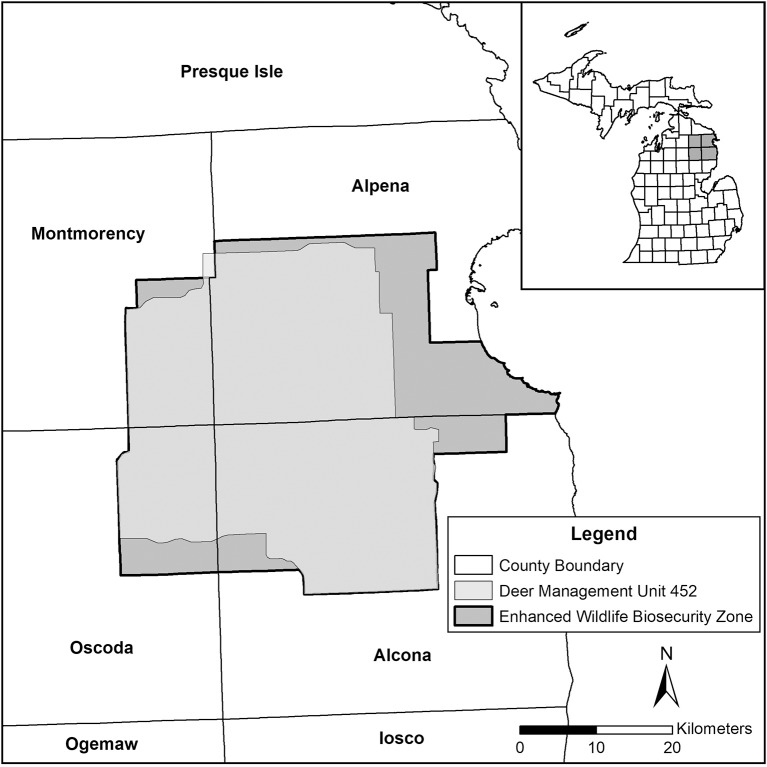
Area of endemic bovine tuberculosis infection in both livestock and wildlife in Michigan, USA, often referred to as the “4-county area” or Deer Management Unit 452 (149,018 ha). The Enhanced Wildlife Biosecurity Zone is an area with increased disease mitigation efforts focused on separating cattle and white-tailed deer (*Odocoileus virginianus*).

Movement of cattle from the MAZ must originate from a bTB accredited-free herd or one that has had a negative whole herd test within the previous 12 months and requires a movement certificate, unless the cattle are being moved directly to slaughter. On March 21, 2018 a new TB Zoning Order was signed into effect by the Michigan Department of Agriculture and Rural Development (MDARD) that established the Enhanced Wildlife Biosecurity Area (EWBA; an area slightly larger than Deer Management Unit (DMU) 452 in the center of the MAZ) (6). Development of the EWBA and increased disease mitigation efforts were an intensified effort to avoid another spike in incidence of infected herds like was seen in 2016 when 4 beef herds, 1 feedlot, and 1 dairy herd within the MAZ were found bTB positive (see Figure 1) (5, 7). As such, if the incidence of bTB infected cattle herds continues to rise or fluctuate like it has in recent years, there is a chance that the 4-county MAZ status or even statewide status (TB Free) could be in jeopardy (5).

### History of bTB in deer in michigan

**Figure 2:**
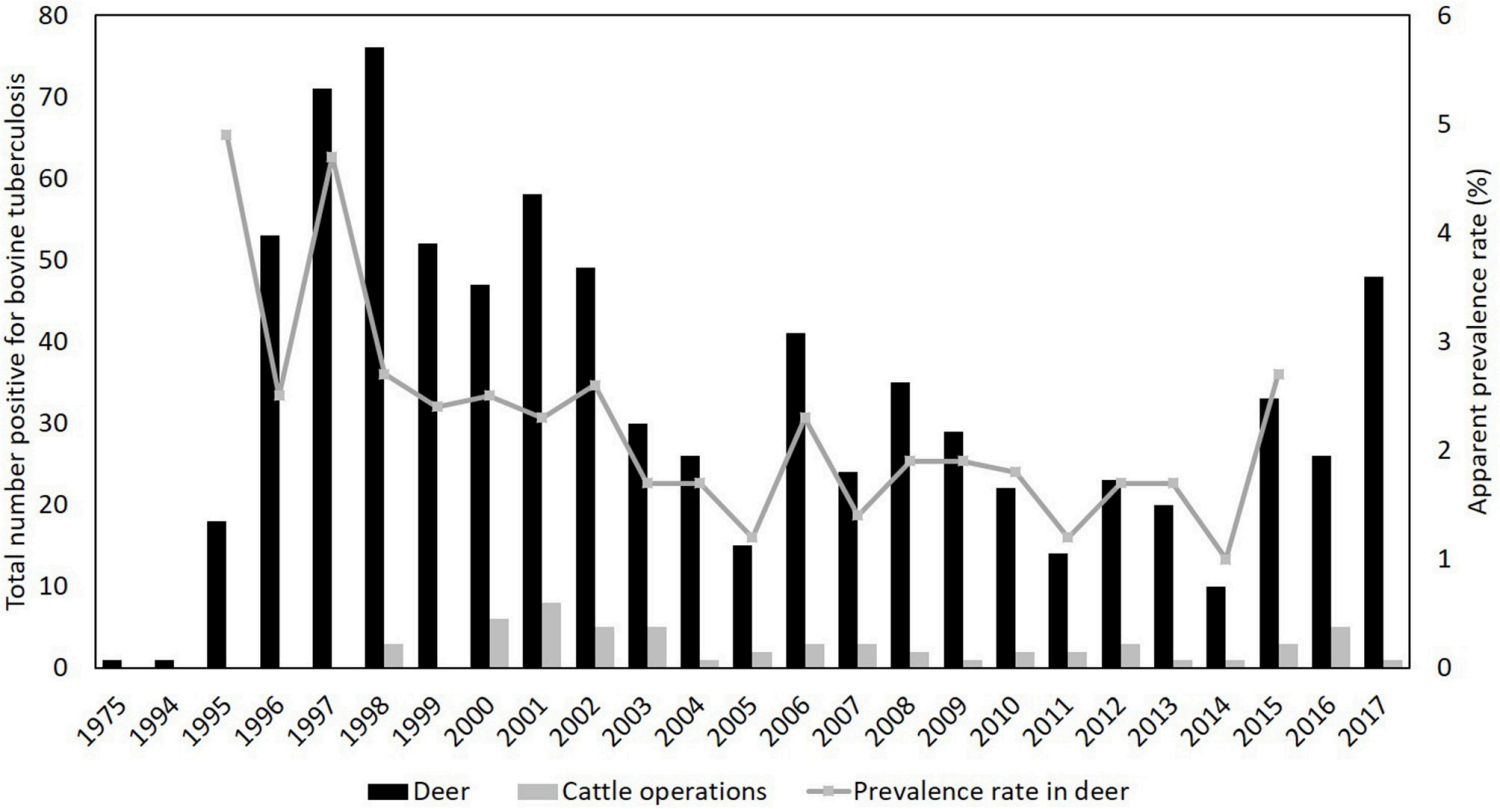
Apparent prevalence rates in white-tailed deer (*Odocoileus virginianus*) as well as numbers of cattle operations and white-tailed deer confirmed positive for infection with *Mycobacterium bovis*.

In 1975 and again in 1994 bTB was detected in white-tailed deer in the northeastern lower peninsula (NELP) of Michigan. After which the Michigan Departments of Natural Resources (MDNR) initiated a surveillance program of testing hunter-harvested deer (8–10) (Figure 2). A collaborative effort was initiated in 1996 by Michigan Departments of Agriculture (MDA), Community Health (MDCH), MDNR, the USDA, and Michigan State University (MSU) to manage bTB by initiating the Michigan Bovine Tuberculosis Eradication Program (11). In 1997, bTB was identified in the first positive cattle herd in the core disease outbreak area since 1974 (12) (Figure 2). In January 1998, the Governor of Michigan directed the MDCH, MDA, and MDNR to develop a plan for eradicating bTB from Michigan deer (13). In summary, the directive included the following components for the 5-county endemic area: (1) implement a deer feeding ban, (2) develop deer harvest quotas consistent with eradication goals, (3) develop methods for eliminating contact between cattle and deer, (4) continue surveillance and determine actual prevalence and evaluate trends, (5) educate stakeholders on managing deer with the goal of eradicating bTB, and (6) enlist a Coordinator to implement the eradication strategy (13). The directive was prepared based on the prioritization of public health and natural resources and insuring the vitality of agricultural industries.

Cattle are acknowledged to be the original source from which bTB or more specifically, *M. bovis* bacterium were disseminated into the spill-over host, deer, which now spill the pathogen back over to cattle (3, 14). The deer in this area of Michigan, then, are acting as a maintenance or reservoir host sustaining the disease on the landscape (see Figure 2) (3). Likelihood of maintaining disease would be increased if there was continued spillover from another reservoir host, such as the original source, cattle. Though considerable attention is paid toward protecting cattle and their feed and water sources from potentially infected wildlife species, it must be emphasized that deer are at risk of infection from cattle as well (3). As bTB-positive livestock operations are identified every year, more novel and aggressive approaches will be required to eradicate bTB from the NELP of Michigan, USA.

The infected deer population of the endemic area contributes to continued infections in cattle (1, 3, 10). This area lies within state-designated DMU 452 which is within a 4-county area consisting of Alcona, Alpena, Montmorency, and Oscoda counties. By 1994, the estimated deer densities where bTB occurred were at or beyond biological carrying capacity (19–23/km^2^) and there were high densities maintained largely through supplemental feeding by hunters and other deer enthusiasts (Figure 2) (15). Apparent prevalence rates for bTB in deer in the endemic area as of 2011 ranged from 1.2% (2005) to as high as 4.9% (1995) and has hovered just below 2% over the two decades since (12). Although apparent prevalence rates are an imperfect predictor, they are frequently the best information available to monitor trends in disease (16). From 1994 to 2009 apparent prevalence of bTB in deer correlated with deer population estimates in the endemic area very well (Figure 2) (12).

### History of baiting and feeding relative to maintenance of bTB in deer in michigan

In general, the bTB endemic area of Michigan consists of several land management types that are relevant to perpetuating the disease and managing the situation: first, several large privately owned parcels of deer habitat are managed exclusively for hunting (17); second, large tracts of public and privately owned forests exist in multiple successional stages thus providing ample deer habitat components in proximity to one another (18); and third, interspersed agricultural lands consisting of dairies, crops, pastures, and beef cattle operations. The makeup of these agricultural lands provides high quality deer habitat in the region.

Supplemental feeding to sustain and concentrate deer and baiting to attract them to specific locations for hunting purposes were common practices in this area and contributed largely to high deer densities and disease transmission (17, 19–21). Prior to restrictions and bans on feeding and baiting, 72% of non-resident and 87% of resident hunters in the NELP of Michigan used bait while hunting (22), illustrating how prevalent these practices had become. Feeding and baiting helped develop a deer population that ultimately exceeded an estimated 20 deer per km^2^ (8). As discussed by 8 baiting and feeding are recognized by natural resources professionals as the primary reasons originally enabling deer to become reservoir hosts for bTB in this area.

## Key concepts in moving from management toward eradication

Approaches to eradicating disease are situational dependent though often include common key components. Components of previous eradication strategies include: (1) implementation of mitigation measures to protect against transmission of *M. bovis* to and from livestock, (2) implementation of management strategies to reduce prevalence in host species including wildlife and livestock; (3) establishment of well-defined goals, plans and policies; and (4) initiation of strategies to build and maintain support of the broad array of stakeholders.

### Current efforts toward eradication in michigan

As in most disease eradication situations, any single strategy alone will rarely eliminate the disease, especially when there are more than a single reservoir host and free-ranging wildlife are involved (23). As such, a combination of strategies need to be implemented in an integrated approach as this will improve efficacy while reducing overall effort and cost (24). In 2008 MDARD initiated the Wildlife Risk Mitigation Project (WRMP) which focused on enrolling and assisting livestock producers in implementing and maintaining an array of measures to reduce risks for transmission of *M. bovis* between deer and cattle on their properties (2, 25–27). Producers were encouraged to participate in the project which entailed education, completing an on-farm assessment of risks, committing to a formal action plan, initiating the action items within the plan, and passing a verification visit to ensure they implemented the plan (25).

A primary risk of transmission between wildlife and cattle stems from shared resources like food, water, and habitat (19, 27–29). Thus, mitigation measures were directed at protecting resources that are concentrated such as stored cattle feed, watering systems and areas routinely occupied by cattle (2, 26–28). It was also recognized that commonly used farm management practices needed to be evaluated and improved upon. Practices such as the collection of waste slurry from cattle that is then applied to crop fields is questionable especially when there's potential for *M. bovis* to be present (30, 31). This practice often occurs during spring green up when nutritionally stressed deer are dispersing from winter concentration areas in search of nutritious food sources like crop residues and lush new growth emerging in crop fields following snow melt (32).

At the initiation of a plan within the WRMP, landowners meet with an agency wildlife biologist on the farm to assess risk factors for disease transmission. Mitigation measures ranging from strategic feeding practices to constructing feed storage facilities are then recommended based on identified risk factors. The WRMP is a science-based program and the efficacy of many of the recommended mitigation strategies have been supported by research findings including the use of fencing (26, 33) and gates (34, 35) to protect stored feed and feeding areas. Risk mitigation strategies prescribed included, but were not limited to: (1) protecting cattle feed by storing it in buildings or within deer-proof fences with gates closed, (2) feeding cattle daily and away from deer cover, (3) strategically positioning water sources to minimize access and potential contamination by deer, and (4) using disease control permits (DCPs) to reduce antlerless deer numbers on and around farms (5). The majority (545 of 620; 88%) of the farmers in the 4-county MAZ participated in the Program and were subject to annual inspections to insure compliance and maintain their verification (5).

When motivation for deer to access food and water is elevated, such as during late winter, increased vigilance and additional measures to exclude or deter deer may be required (26, 36). The efficacy of mitigation measures is directly related to the motivation of an animal to overcome it and the vigilance of the farmer. Motivation also varies with circumstances relative to season (e.g., severity or length of winter, drought conditions in the summer) and availability of natural foods and water. Producers must be cognizant of these factors, and therefore, when risk is increased, must increase vigilance to maintain an effective level of biosecurity (26, 36, 37). Such mitigation measures and environmental influences are discussed during risk assessments to insure producers understand that wildlife risks are not static and identify factors and scenarios that may increase risk.

#### Current efforts: exclusionary fences

The use of fencing to exclude deer is an effective means for protecting concentrated resources meant for livestock (2, 26, 33). Numerous fence types exist and fence selection can be based on the predicted level of motivation for deer to breach, the desired longevity, and associated cost (33, 38). In high-biosecurity situations where essentially no deer breaches are acceptable, woven-wire fences ≥2.44 m in height are recommended (33, 39). Interestingly, the “weakest link” of a fence is the gate, which obviously must be closed to be effective (26, 34). While this may seem like common sense, in areas where frequent access is needed, livestock producers commonly become lax, leaving gates open, especially during daylight hours. Deer, then, have been documented entering fenced areas of stored feed through open gates in the middle of the day when it was assumed they would not be nearby or active (26).

#### Current efforts: livestock protection dogs

Livestock protection dogs (LPDs), traditionally developed and used for reducing the killing of livestock by predators, have also proven effective in keeping deer from directly and indirectly coming in contact with cattle (40). Using specially trained dogs for protecting numerous agricultural resources is becoming more widespread (41, 42). For example, LPDs have proven effective in protecting crops (43), cattle pastures, and feed (40, 44). In the case of transmission of *M. bovis* between cattle and deer in which concerns over indirect transmission through contaminated resources are greatest, LPDs employed to protect stored feed and other resources would be beneficial (40, 44). Although LPDs can effectively repel deer to protect localized areas and livestock, there is a point in which the size of the area or the amount of deer activity exceeds the abilities of a single LPD and either additional LPDs or integrating other measures such as exclusionary fences are needed (33, 44).

#### Current efforts: strategically locating feed and water for cattle

Currently, 88% of commercial farms in the MAZ are incorporating practices focused on protecting cattle-related resources from wildlife that is potentially harboring bTB (5). Although participation is high, increased emphasis on consistent use and maintenance of mitigation measures is needed (26). Such resources include water, feed, and mineral supplements, all of which are sought by deer and other wildlife and should be a focus of concern regarding the transmission of *M. bovis* (19, 28, 45, 46). Initially, USDA cost-share programs assisted producers in incorporating secure feed storage options including hoop barns and deer-exclusionary fencing to minimize deer access to cattle resources. Refined feeding strategies including limiting provisions to just what a group of cattle will consume that day and constricting the time and duration of availability to just daylight hours can help reduce deer activity in cattle feeding areas (5). Water, though, needs to be available continuously so could be more difficult to protect from contamination by deer (5). Storing and providing cattle resources (feed, supplements, minerals, water, etc.) away from permanent deer habitat and closer to areas of human activity is also recommended.

#### Current efforts: cattle identification and tracking

Annual whole-herd testing of cattle for bTB and outfitting cattle with radio-frequency identification (RFID) tags became a requirement for Michigan producers in the endemic area to move live cattle off their farms in 2007. These requirements enable trace-back investigations to locate where and when bTB-infected cattle shared the same space as other cattle, with the goal of identifying other potentially infected animals and premises (29, 37, 47). Although the infection of a herd due to movement of an infected cow into that herd occurs (48), it was presumed to be a lesser risk for cattle producers in Michigan than infected deer (29, 47). Yet recent cases outside of the endemic area and within the accredited-free zone of southern Michigan suggest spread of bTB via infected cattle may actually be increasing (49).

#### Current efforts: reducing deer numbers

Population reductions are often considered or used in response to outbreak of disease and involves reducing the density of the host population through strategic lethal removals, usually through culling by professional sharpshooters, or increased recreational hunter harvest (50, 51). Large-scale removals of reservoir species have been implemented and proven effective in some cases (23, 52–55). Though used to a degree in the endemic area of Michigan, these options have proven controversial and have not been wholly accepted by producers, hunters or other publics in Michigan (24, 56).

With the goal of reducing the potential for transmission of *M. bovis* between deer and cattle the MDNR initiated a program in 1998 in which cattle producers could acquire DCPs allowing them to personally address the deer situation on their land by harvesting deer themselves or enlisting the help of sharpshooters with the USDA Wildlife Services (12). Producer use of these permits, though, was low. Only 12% of 6,427 tags were filled in 2008 and deer numbers have since increased as have associated disease prevalence rates (12, 57).

Damage tags or block permits were also available to producers who were experiencing damage to crops by deer, allowing them to harvest deer on their property to alleviate ongoing problems (57, 58). Similar to DCPs, participation was low and lack of local public support was presumed to be the cause (12, 57, 59). Occasionally, negative concerns about these non-traditional deer harvest strategies were voiced by owners of recreational lands adjacent to at-risk farms (12). For example, even when deer density estimates were 8–15/km^2^ and crop damage was substantial, only five of 31 alfalfa growers participating in a crop damage project requested permits to control damage on their property and only 42% of issued permits were used. Similarly, red kidney bean growers were issued a total of 88 permits and only 23% were used (60, 61). These data illustrate that even when landowners were faced with substantial amounts of crop damage and provided permits to reduce deer numbers, they were not using them (60, 61).

The MDNR increased the number of available deer tags and the number of hunting seasons, with the focus on removal of antlerless deer, and successfully reduced deer numbers within DMU 452 by 50% from 1995 to 2004 (12, 62). However, deer numbers rebounded rapidly since 2005 to >110,000 and remained steady through 2009 (12). More recently hunting opportunity and harvest potential has been essentially unlimited in DMU 452, though hunters have harvested less than one thousandth of the tags available (i.e., 4,388 deer from 5,575,390 potential tags), demonstrating that demand for opportunity has been saturated (63). Although the MDNR was effective in reducing deer numbers initially, hunters were not overwhelmingly supportive of these actions (22). As a new strategy to address insufficient harvest on farms, it is now a requirement for livestock producers to include and implement a deer reduction component within their EWB Plan, specifically focused on those deer routinely in proximity to farms with cattle (5). As this is a recently enacted requirement, the effects are yet to be seen.

### Intensifying efforts to reduce potential transmission of *M. bovis*

Nearly 20 years ago it was stated that “The measures of apparent bovine TB prevalence have decreased by half since 1997, providing hopeful preliminary evidence that eradication strategies are succeeding” (15), but bTB still persists in Michigan. Ongoing and increased efforts to reduce the persistence of *M. bovis* continue; however, the rate of cattle operations being identified as positive for bTB fluctuates at levels that put the accredited-free status of Michigan in jeopardy (>3 positive herds detected/year) (5). Despite extensive efforts prescribed by the previous WRMP, bTB-positive herds continue to be identified each year, thus new approaches are needed if the goal is still to eliminate the disease. To this end the EWB Project was initiated to involve more thorough on-the-ground assessments of properties housing cattle by an “EpiTeam,” similar to what is used following the detection of positive cattle. Each team includes a MDARD veterinarian, a USDA or Alpena Conservation District wildlife biologist, a MSU Extension cattle specialist, and a local producer (5). Each assessment results in an action plan (now entitled “Enhanced Wildlife Biosecurity Plan”) that needs to be implemented on the ground by the producer, similar to how the WRMP was implemented from 2008 to present. Producers must implement and maintain all prescribed mitigation measures relating to high-risk areas on their farms by December 31, 2019 or will lose their ability to sell cattle other than directly to slaughter (6).

#### Intensified efforts: targeted deer removals

All existing 130 commercial cattle producers in the EWB Area require a deer removal component that enables sharpshooters to remove deer from in and around farms and pastures. For example, in Dressel's (32) study up to 13 deer were removed from a single landowner's property. Action plans in the EWBP are designed to eliminate deer whose home range includes farms and deter others from establishing ranges in proximity to farms (5). The frequency of visitation to highly-desirable resources such as stored feed and agricultural crops may be a learned behavior that could be curtailed by removing mature does or entire family groups of offending deer (64). It has been documented that fawns can learn movement patterns from adult does and that it is typically a few specific deer in a given area that will share space and time with cattle or frequent stored feed areas (27, 36, 37). As such, targeted removal of offending individuals may curb present and future visitation of farms by deer. Research has shown deer frequent farms the most often during: January through mid-April; and Mid-July through August (26, 36), thus these periods are when removal efforts should be focused.

#### Intensified efforts: strategic habitat manipulations

Wildlife management consists of three components: (1) the biota or populations, (2) the habitats or ecosystems organisms need to persist, and (3) the people or stakeholders that live in the ecosystems and interact with the wildlife resource (65). To date, bTB research and management practices have been directed primarily at two components, namely the biota (i.e., deer) by reducing numbers through recreational hunting and targeted deer removals and the people (i.e., hunters, producers) by manipulating harvest regulations, deer baiting, and feeding practices, and how producers store and protect feed and water resources. Historically, the third component of wildlife management, the species' habitat, has not been factored into bTB management strategies. Perhaps because, as Felix et al. (66) suggested, “managers may lack sufficient understanding of long-term spatial and temporal links between habitat supply and population response.” There has, though, been an extensive amount of research on why and how forest management practices can be used to enhance or reduce quality of deer habitat [e.g., (67, 68)] and potentially influence the distribution of deer across a landscape (18).

The Alpena-Montmorency Conservation District of Michigan has recently initiated a cost-share program that may assist in influencing deer movement patterns, potentially away from stored feed, water sources and livestock concentrations, by initiating habitat improvements for deer through forest management activities. This new program, if implemented strategically could stimulate deer to redistribute themselves away from agricultural areas to other, naturally occurring vegetation types. The program also includes incentives that encourage data collection and the liberal harvest of antlerless deer (69).

The quality and distribution of a species' habitat is a primary driver influencing the spatial and temporal distribution of species [e.g., (70)], including deer [e.g., (18, 71)] and elk [e.g., (72, 73)]. Recognizing how habitat quality and its distribution can influence the movement patterns of a species, a strategy could be to use this basic ecological principle as a tool to combat bTB in the NELP. An additional step may be to take measures to lessen the quality of habitat for deer on and adjacent to farms, lowering the area's carrying capacity, the desire of deer to be there, and the fitness of deer that persist.

The core of the bTB area, DMU452 is composed primarily of private land (93%) (74) that is dominated by forest cover types. For example, within Alpena County alone, 60% of the area is covered by lowland conifer swamps and northern hardwood forests interspersed among agricultural areas. Much of the forest is relatively later successional stage, especially on private lands. Given that the life requisites of deer in this area include: spring and summer food, thermal cover, and fall and winter food (66), much of the agricultural lands and livestock areas are often under tremendous feeding pressure by deer especially in late-winter through summer (32, 60, 61, 75). A cover type lacking in this area that deer could use extensively for feeding and cover is regenerating deciduous stands (e.g., aspen clearcuts of predominately early age classes) (66). Experimenting with forest management practices as a method to manipulate how deer use the landscape has merit.

Habitat management on public lands is a primary activity used by agencies to meet wildlife management objectives and satisfy a diversity of stakeholders, yet it is poorly understood how frequently or what types of management, if any, occur across private lands. The use of landowner incentive or cost-share programs to manipulate forest cover types to improve habitat conditions away from agricultural lands and livestock operations should be investigated for their efficacy in: (1) providing quality habitat, (2) shifting the distribution of deer away from agricultural areas at high risk for transmission of *M. bovis*, (3) reducing crop damage, and (4) meeting economic objectives of landowners for harvesting forest types. Such a habitat-based bTB management and research approach could be initiated and simultaneously integrated with other bTB mitigation practices. The successful management of this complex problem could be enhanced if the habitat for deer were factored into the management equation.

### Potential future efforts

Original actions to eradicate bTB in Michigan combined with recently emerging science-based strategies have all been insufficient to date, primarily due to waning stakeholder support. Several new strategies and directions are mentioned above and have begun, here we discuss additional potential measures to consider if the collective desire of agencies, stakeholders, and other publics is to eradicate bTB from Michigan.

#### Potential future efforts: reducing deer numbers

As stated by Riley et al. (76), “An assumption in most conventional deer harvest strategies is that adequate demand for and successful use of antlerless deer permits exists to achieve desired deer harvest.” As deer densities decline and number of deer encounters are reduced, hunter perception and support, effort, and desire to continue hunting fade and hunters will often transition to other locations or species (77, 78). When hunter harvest is no longer effective in maintaining deer populations at or below goal, additional measures must be contemplated. In such situations “Hunting eventually may become less a recreation and more a community service or civic duty … Culling may be a more appropriate term for the kind and purpose of hunting under such circumstances” (76). Although recreational hunting is and should remain the primary means for managing white-tailed deer, there are situations in which it may not be safe, feasible, or effective and other means need to be considered (79). Within DMU 452 where deer reductions are needed and current harvest is insufficient, strategies like earn-a-buck or incentivizing hunters by allowing easy donation or profiting from venison may be worth consideration (79–81).

Most (>90%) of the bTB area in Michigan is privately owned (74) which has contributed to challenges in achieving wildlife management goals (9). Although purely speculative, it is uncertain about what the future for large privately-owned “hunt clubs” will be with consistently declining numbers of hunters. Will the owners of these lands want to hunt them in the future or use them simply as family get-aways? How will this affect the local deer population? A decreasing trend in hunters has been well-documented in the US (82, 83) and in Michigan specifically (84, 85). Because of these trends, other approaches might be warranted such as the MDNR purchasing large tracts of hunt clubs or other private lands (farms) to improve access. For example, from January 1998 to November 2018, the MDNR purchased a total of 34,240 ha state-wide with an average of 1,630 ha being purchased annually and the mean amount of land acquired per transaction was 53 ha (K. Wildman, Biologist, MDNR, personal communication, 05 Nov 2018). Non-profit conservation organizations such as the Rocky Mountain Elk Foundation are often partners in purchasing land which the state then manages and oftentimes provides public access for hunting. A local example in northern Michigan was the purchase of the Green Timbers tract in 1982. This property is now attached to the Pigeon River Country State Forest and provides unique walk-in only hunting and other recreational activities (e.g., backpacking, hiking, cross country) for the public. Acquisitions such as this improve the ability of the MDNR to manage the deer population and provide opportunity to its constituents.

#### Potential future efforts: vaccination program for deer

An additional novel tool that could aid eradication of bTB in Michigan is an oral vaccine against bTB for deer. Interest in using a vaccine for bTB in deer is increasing (32, 63, 86). Bacille Calmette-Guerin (BCG) vaccine reduces disease severity by decreasing gross lesions and sites of infection, suggesting potential for reducing transmission and minimizing endemic infection in wildlife (87, 88). Significant progress has been made in demonstrating the safety, efficacy, and feasibility of implementing a vaccination program against bTB for deer (89–92). Researchers modeled vaccination and demonstrated that vaccinating just 50% of the deer would contribute to an 86% probability of eradicating bovine tuberculosis in DMU 452 in 30 years (63). Interestingly, in the presence of recreational baiting it would be highly unlikely to achieve eradication within the next 30 years at the same vaccination rate (63). A vaccination rate higher than 50% could likely be achieved based on an experiment where placebo vaccine baits were effectively delivered to free-ranging deer (32) which would increase the probability of eradication. Of course, implementing a vaccination program while maintaining the use of additional management strategies; restrictions on baiting, liberal recreational harvest, DCPs, and fencing stored feed and other cattle resources would be the most efficient path to eradication (63, 86).

#### Potential future efforts: novel diagnostic tests for bTB

As current live-test methods involve multiple animal handlings, take 48–72 h to produce results, or require specialized laboratory procedures, improved methods are needed for reliable and timely detection of bTB (93, 94). A “trap–test–cull” project was evaluated using a rapid test and live capture of deer, though it was deemed cost-prohibitive (>$1.5 million US annually) and ineffective in reducing prevalence of bTB (95). Recently developed methods that enable the antemortem detection of unique biomarkers of disease suggest improved diagnostics are becoming available. For example, infection by *M. bovis* results in the presence of specific peptides in the blood which can be detected with common laboratory analyses (96). Additionally, the analyses of breath from cattle to detect bTB-specific volatile organic compounds has proven effective in experimental settings and has potential for applications with deer (94). Also, genotyping particular strains of bTB pathogens enable back tracking to determine the source herd of cattle for the disease (97). New tools like these and the support to develop them are desperately needed.

### The people piece

Public support and involvement is essential if complete eradication is the goal. Are Michigan residents accepting of a low level of bTB sustained in their deer herd? It was apparent in 2006 that Michigan hunters felt bTB was not a problem, ranking it considerably lower than “more extensive problems” including too few mature bucks and too few deer in general (12). Are Michigan livestock producers comfortable with the risk that they may have a reactor cow in this year's whole-herd test and that theirs could be the next positive herd? It is clear that Federal and State agricultural agencies are losing tolerance for reoccurring positive cattle farms. As it should be, input from stakeholder groups and various publics have played a large role in political and management decisions regarding bTB in Michigan since 1994 when the second bTB positive deer in 20 years was found. There is potential that had managers been more empowered or convincing and decision makers more stalwart the bTB situation in NELP may be quite different today. Despite extensive surveys examining strategies used to improve stakeholder appreciation of the situation with bTB in deer (98–101), public and political support has been too little to enable the actions necessary to improve the situation (101). To make better progress going forward, more emphasis must be placed on the human dimensions aspects of the issue by more effectively engaging the diversity of stakeholders associated with this deer-bTB-agricultural industry issue.

#### Policy based on science or public demand?

Although state wildlife management agencies are responsible for managing wildlife populations, habitats, and the people who use wildlife resources (65), elected and appointed government officials typically make the underlying decisions driving management actions of agencies (102). In 1996, Michigan voters elected to transfer the responsibility for managing game animals from the MDNR to the 7-member governor appointed Natural Resources Commission (NRC). The NRC was mandated to integrate scientific findings and public input into new policies that the MDNR follows; in turn, the MDNR provides recommendations to the NRC to help them make informed decisions when establishing such policies (20). Policy established by the NRC in 2007 presents the goals of the MDNR as using science-based management practices to maintain a healthy deer population as determined by the carrying capacity of its range and the effects upon native plant communities, crops, and public safety (103). Additionally, they set out to maintain an active educational program to inform the public on practices of deer management for achieving a healthy and vigorous herd (103). Despite these basic, well-intended goals driving policy, public trust (of NELP residents) in the ability of MDNR to set deer hunting rules relative to eradicating bTB was lower than 50% in 2011 (104). This distrust has impacted the ability of MDNR to manage bTB and created backlash by local residents and hunting constituency groups (105).

Tools such as spatial models for forecasting likelihood of disease eradication given various approaches are the types of informative tools needed to aid in establishing goals and creating policy (63). A key strategy for facilitating scientifically based decisions leading to effective management actions lies in providing policy makers with accurate information derived from high quality research while respecting their role of representing those that elected or appointed them (102). Further, educating the general public and earning acceptance and trust are also essential to successful management of healthy wildlife populations and their habitats (15, 22).

#### Building widespread stakeholder support

Initial efforts by state and federal agencies to eradicate bTB in Michigan were extensive despite minimal public support (106). To be effective and successful, actions initiated by agencies have to be accepted and adopted by citizens including hunters, livestock producers, and wildlife viewers. For example, MDNR initiated strategies to reduce deer numbers through increased availability of hunting licenses and implemented baiting and feeding restrictions (20, 56). Public support and action was needed to harvest additional antlerless deer and to cease baiting and feeding. Although there was a documented 50% decline in apparent prevalence from 1995 to 2004 due to reductions in deer numbers and restricting baiting and feeding (107), deer numbers and prevalence rates have since rebounded. As demonstrated by the incessant reappearance of bTB in deer and cattle, it is apparent public support and involvement are essential for successful eradication or even tempered control (20, 106, 108). It is also apparent that the lucid presentation of specific disease-related risks to one's personal interests are needed to truly bring about action and change (99, 100). Frequently updated information with an emphasis on successes is essential to maintaining or increasing stakeholder support (98).

In addition to insufficient stakeholder support, there has been decreasing financial support to and from federal and state agencies to enable the eradication of bTB from wildlife and livestock in Michigan. This issue has led to fewer personnel and waning awareness and support from most publics. Thus, current and future efforts toward eradicating bTB require maximizing knowledge gained from past efforts to inform next steps for research and management (62). To this end, modeling efforts have helped predict likely outcomes given the tools and resources available to begin answering questions to help optimize and select combinations of strategies to implement (63). Without incorporating new tools and revising strategies, it was predicted that eradicating bTB from Michigan in the next 30 years was unlikely (63, 95).

It has become clear that ongoing strategies for eradicating or even minimizing the transmission of bTB in Michigan have been insufficient, primarily due to lack of sufficient long-term determination of stakeholders. If the Michigan and US goal is to protect the entire country's cattle herd and trade status, increased support and strategies are needed. Further, it is apparent that increased public acceptance and involvement will be required to defeat the challenges associated with the eradication of bTB (56, 107).

Unfortunately, these challenges are deeply rooted in the culture of the area and will not be overcome easily. There are apparent divides and disconnects amongst the interests and demands of various factions of the public (i.e., hunters, cattle producers, policy makers, general public), with public servants from natural resource and agricultural agencies struggling to regain healthy wildlife and livestock populations for them. It seems that through efforts to achieve healthy deer densities in Michigan following the appearance of bTB, public resentment has actually grown (12, 62). Agencies need improvements in public outreach about all aspects of the bTB issue to reverse this trend and garner support for the intentions behind management actions. Given the current popularity and user involvement in social media (i.e., YouTube, Instagram, Podcasts, etc.), it is a new tool that could be used to aid ongoing and future efforts associated with bTB. Although previous efforts to engage and motivate hunters to actively participate in non-traditional deer management actions (i.e., increased harvest of antlerless deer) failed over the long term, significant changes such as providing extended or alternative seasons and increasing attention on new hunters may improve participation (101). Unfortunately, common trends such as managing for more, larger, and more mature (i.e., older) male deer on the landscape, primarily through imposing antler point restrictions, does not align well with disease management strategies focused on removing more males with an emphasis on older age classes (10).

### Optional approaches toward managing bTB in michigan

Going forward, agencies need to (1) establish long-term, mutually agreed upon objectives, (2) develop well-defined strategies that align with those objectives, and (3) develop and implement practices to evaluate the efficacy of those strategies (109). All options toward managing disease, including no action, need to be considered in establishing objectives (24, 86). First and foremost it needs to be determined what the long-term goal is: status quo, eradicating bTB throughout Michigan, eliminating bTB in deer in Michigan, or eliminating bTB in cattle in Michigan. If the presence of bTB in Michigan truly is acceptable, there is always the option of no additional management action whatsoever, although this may need to be coupled with the buyout of all cattle across the region to eliminate potential for cattle becoming infected. Additionally, compartmentalization could be considered to limit the potential for geographic spread of bTB through the use of significant barriers such as large-scale exclusionary fences for deer (24). It was well-stated by Olmstead and Rhode (110) regarding the interconnectedness of the cattle industry, “Given the benefits from trading in livestock and the contagious nature of the disease, it was more efficient to build a “fence” around the entire country than to create barriers around each and every farm.”

If the goal is still to eradicate bTB across Michigan as stated by the Governor in 1998, then the potential exists to make great strides. Actions should include but are not limited to: significantly reducing deer densities with focus on those in the vicinity of cattle operations, eliminating baiting and supplemental feeding, segregating wildlife and cattle/cattle resources, using habitat management to change the spatial distribution of deer, and deploying a vaccine for deer.

If the goal is only to eliminate bTB in cattle, the strategy is relatively straightforward especially if all transmission is occurring only between deer and cattle (111). With cattle being the primary concern, excluding deer from all cattle-related resources with true deer exclusionary fencing (i.e., 2.4-m-h woven wire fence) is needed (24, 26, 39). Where this is not possible, such as a body of water bordered by cover used by deer and cattle pastures, either the deer or cattle must be excluded. Although reliable deer-exclusionary fence is initially expensive and may be considered unsightly, it is effective when maintained and would minimize potential for transmission via indirect and direct contact (24, 26, 33, 38). This level of biosecurity is commonplace in other production animal systems such as within the swine industry (24, 112, 113), especially in areas where the threat of disease transmission is a reality. Permanent deer-proof fences are also commonplace and widely accepted in areas where the captive cervid industry is active, as well as along expansive stretches of highway systems throughout the US where deer-vehicle collisions had been common. These fences are also used around the world in places such as in Africa because they enable managers to achieve extensive and reliable manipulation and protection of various species (33). Given the serious nature of eradicating bTB, reliable management of deer and cattle are needed in Michigan and thus similar measures could be considered.

## Conclusion

The ongoing situation with bTB in Michigan has been a persistent and expensive management challenge for livestock producers and state and federal agencies for more than a quarter of a century. As biologists and public servants, we may feel ethically committed to ridding the landscape of this disease that impacts the wildlife resource and a primary agricultural industry. But unless the societal and related political support for this exists, perhaps we need to either stand down or double down. The situation in Michigan is a multi-faceted issue with several imposing barriers, ecologically and socially, that are impeding the possibility for progress toward eradicating the disease. The first and foremost challenge is inadequate public concern over the health of the deer population and cattle herd and subsequent lack of political support and action. This challenge obstructs many crucial steps in wildlife management toward eradication, including the banning of baiting and feeding, reducing host populations, and understanding and accepting the severity of the bTB situation across the landscape.

If there was increased public concern about the occurrence of bTB in wildlife, livestock, and humans there would likely be compounded support and participation in actively pursuing eradication. As demonstrated during the era of market hunting, even before the advent of modern hunting tools and technologies (i.e., high-powered rifles and scopes, night vision, remote cameras, helicopters, drones), Americans demonstrated our ability to severely reduce, and in some cases, decimate deer populations when motivated. Conversely and more recently, due to changes in motivators, we have demonstrated our ability to develop large numbers and concentrations of white-tailed deer. Now we must refocus on maintaining populations of fewer but healthy deer in concert with the limitations of local agricultural goals and available natural vegetation types that can provide deer habitat. In 1949, Aldo Leopold wrote, “A thing is right when it tends to maintain the integrity, stability, and beauty of the biotic community, it is wrong when it tends otherwise” (114). Natural resource professionals can still keep this goal in mind while simultaneously acknowledging and addressing the food production needs of our continually growing and hungry populous.

The toolbox contains much of what is needed to combat bTB in Michigan; including increased hunting license allocations, increased availability of disease permits, financial cost-share programs to increase biosecurity on farms, feeding and baiting bans, the use of educational stakeholder meetings, new novel tools to facilitate diagnosis and surveillance, and even a vaccine for deer or evaluating the use habitat manipulations to redistribute deer. None of these tools will be effective alone, they must be applied aggressively and in unison to complement each other. Progress has been made in understanding and managing livestock-wildlife interactions and the transmission of bTB in the Michigan landscape and recent decisions and new strategies have great potential.

## Author contributions

KV, ML, and HC contributed equally in the development of the idea, collection of the data, and preparation of the manuscript. KV fleshed out the original outline. ML drafted the manuscript and continually incorporated and massaged his, KV and HC's thoughts. All authors continually reviewed and edited the manuscript, producing the submitted draft.

### Conflict of interest statement

The authors declare that the research was conducted in the absence of any commercial or financial relationships that could be construed as a potential conflict of interest. The handling editor declared a past collaboration with one of the authors HC.
